# 1-(4-Methyl­phenyl­sulfon­yl)-2-{[3-methyl-4-(2,2,2-tri­fluoro­eth­oxy)pyridin-2-yl]methyl­sulfan­yl}-1*H*-1,3-benzimidazole

**DOI:** 10.1107/S1600536813031899

**Published:** 2013-11-30

**Authors:** C. M. Shivaprasad, S. Madan Kumar, T. R. Swaroop, K. S. Rangappa, N. K. Lokanath

**Affiliations:** a Department of Studies in Chemistry, Manasagangotri, University of Mysore, Mysore 570 006, India; b Department of Studies in Physics, Manasagangotri, University of Mysore, Mysore 570 006, India

## Abstract

In the title compound, C_23_H_20_F_3_N_3_O_3_S_2_, the benzo­imidazole unit makes dihedral angles of 5.02 (1) and 76.42 (1)°, respectively, with the pyridine and methyl­benzene rings; the dihedral angle between the pyridine and methyl­benzene rings is 72.19 (1)°. In the crystal, mol­ecules are connected by weak C—H⋯F, C—H⋯O and C—H⋯N hydrogen bonds. Weak C—H⋯π inter­actions and π–π stacking [centroid–centroid distance = 3.6485 (14) Å] are also observed. The overall packing shows a three-dimensional architecture. The crystal structure contains a void of 51 Å^3^, but no solvent mol­ecule (hexane or ethyl acetate) is located within it.

## Related literature

For the biological activity of benzo­imidazole derivatives, see: Bansal & Silakari (2012 ▶); Ates-Alagoz *et al.* (2004 ▶). For hydrogen-bond motifs, see: Bernstein *et al.* (1995 ▶).
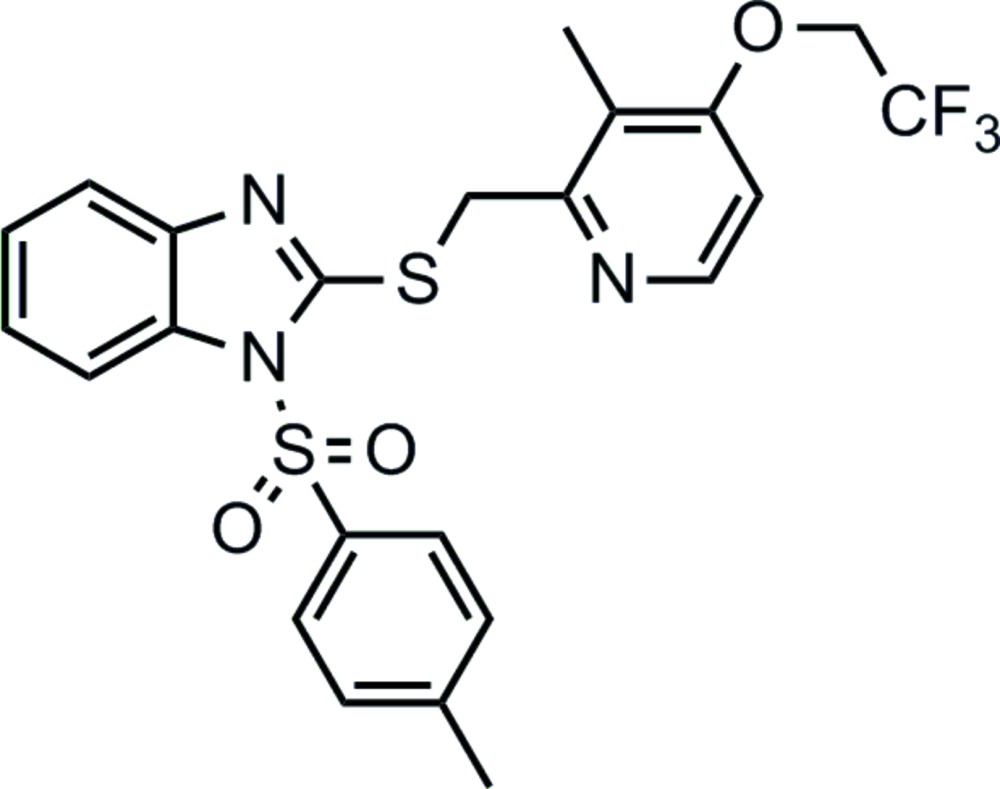


## Experimental

### 

#### Crystal data

C_23_H_20_F_3_N_3_O_3_S_2_
*M* *_r_* = 507.56
Triclinic, 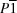

*a* = 9.1129 (6) Å
*b* = 9.5116 (6) Å
*c* = 13.7914 (8) Å
α = 90.978 (3)°
β = 101.749 (2)°
γ = 93.449 (3)°
*V* = 1167.72 (13) Å^3^
*Z* = 2
Cu *K*α radiation
μ = 2.56 mm^−1^
*T* = 296 K
0.21 × 0.20 × 0.20 mm

#### Data collection

Bruker X8 Proteum diffractometer
Absorption correction: multi-scan (*SADABS*; Bruker, 2013 ▶) *T*_min_ = 0.615, *T*_max_ = 0.628
13085 measured reflections
3779 independent reflections
3301 reflections with *I* > 2σ(*I*)
*R*_int_ = 0.049

#### Refinement

*R*[*F* ^2^ > 2σ(*F* ^2^)] = 0.050
*wR*(*F* ^2^) = 0.144
*S* = 1.08
3779 reflections
310 parameters
H-atom parameters constrained
Δρ_max_ = 0.32 e Å^−3^
Δρ_min_ = −0.44 e Å^−3^

### 

Data collection: *APEX2* (Bruker, 2013 ▶); cell refinement: *SAINT* (Bruker, 2013 ▶); data reduction: *SAINT*; program(s) used to solve structure: *SHELXS97* (Sheldrick, 2008 ▶); program(s) used to refine structure: *SHELXL97* (Sheldrick, 2008 ▶); molecular graphics: *Mercury* (Macrae *et al.*, 2008 ▶); software used to prepare material for publication: *Mercury*.

## Supplementary Material

Crystal structure: contains datablock(s) global, I. DOI: 10.1107/S1600536813031899/xu5751sup1.cif

Structure factors: contains datablock(s) I. DOI: 10.1107/S1600536813031899/xu5751Isup2.hkl

Supplementary material file. DOI: 10.1107/S1600536813031899/xu5751Isup3.cml

Additional supplementary materials:  crystallographic information; 3D view; checkCIF report

## Figures and Tables

**Table 1:** Hydrogen-bond geometry (Å, °) *Cg*1 is the centroid of the imidazole ring.

*D*—H⋯*A*	*D*—H	H⋯*A*	*D*⋯*A*	*D*—H⋯*A*
C2—H2⋯F2^i^	0.93	2.49	3.363 (4)	156
C15—H15*B*⋯O3^ii^	0.97	2.52	3.420 (3)	155
C18—H18⋯N3^iii^	0.93	2.60	3.336 (3)	136
C18—H18⋯*Cg*1	0.93	2.83	3.463 (3)	126

Symmetry codes: (i) 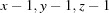; (ii) 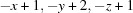; (iii) 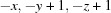.

**Fig. 1.**
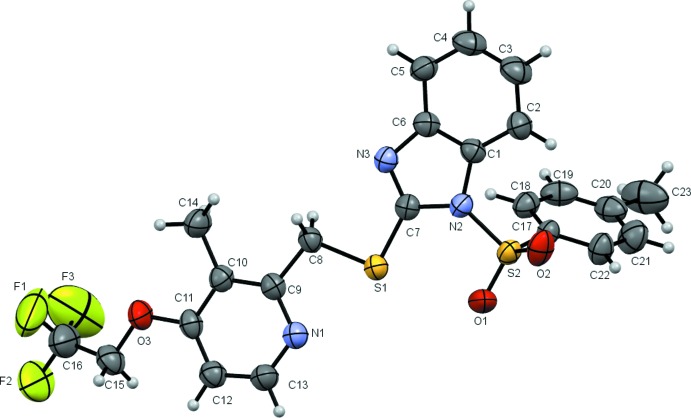
ORTEP diagram of the title compound with 50% probability ellipsoids.

**Fig. 2.**
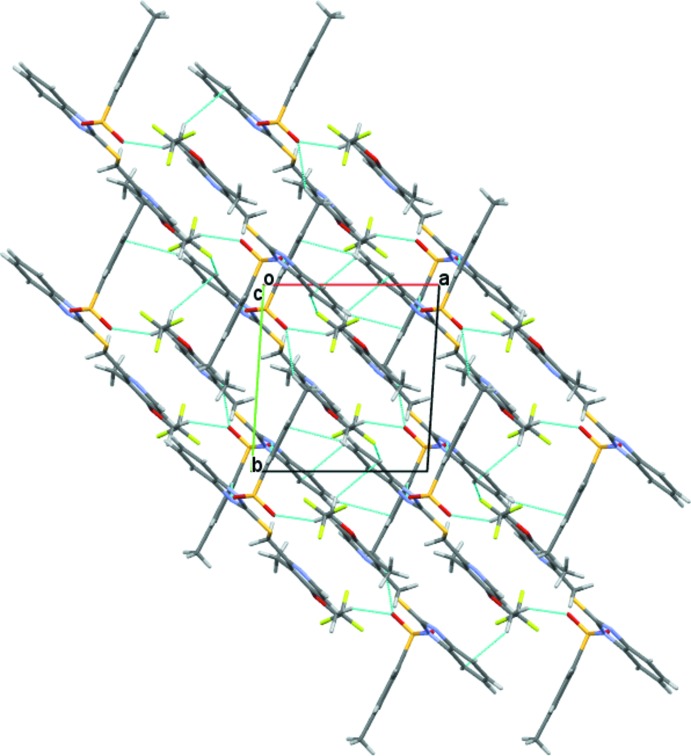
Packing diagram of molecule, viewed along the crystallographic c axis. Dotted lines indicate hydrogen bonds and short contacts involved.

## supplementary crystallographic information

### 1. Comment

The benzimidazole derivatives acts as antioxidant (Ates-Alagoz *et al.*, 2004) and show biological activities (Bansal & Silakari, 2012).

The ORTEP of the title molecules is shown in figure 1. the benzoimidazole moiety (N2-N3/C1-C7) makes a dihedral angle of 5.02 (1)° and 76.42 (1)° with pyridin ring (N1/C9-C13) and phenyl ring (C17-C22), respectively. The dihedral angle betwen the pyridin ring and phenyl moiety is 72.19 (1)°.

The molecules in the crystal structure are connected with C—H···F, C—H···O, C—H···N and C—H···O intermolecular hydrogen bonds (Fig. 2 & Table 1). The C2—H2···F2 forms infinite chains along *b*-axis. C15—H15B···O3 shows R^2^_2_(26) ring motif while C18—H18···N3 shows R^2^_2_(14) ring motif (Bernstein *et al.*, 1995). Also, short contacts C—H···π and π···π [centroid-centroid distance of 3.6485 (14) Å] is observed. Overall packing of molecules depicts three dimesional architecture. All the above interactions of the molecule generates three-dimensional architecture (Fig. 2).

### 2. Experimental

To a solution of 2-(((3-methyl-4-(2,2,2-trifluoroethoxy)pyridin-2-yl)methyl)thio)-1*H*-benzo[*d*] imidazole (10 mmol) and tetrabutyl ammonium bromide (1 mmol) in toluene (20 ml), a solution of 50% KOH (25 ml) was added at 0°C followed by the addition of toluenesulfonyl chloride (12 mmol). The reaction mixture was allowed for vigorous stirring at room temperature for 6 h and the reaction progress was monitored by TLC. After the completion of the reaction, organic phase was separated from the aqueous phase and the organic phase was washed with water (20 mL) and brine (20 mL), dried over anhydrous sodium sulfate and concentrated to give crude product 2-(3-methyl-4-(2,2,2-trifluoroethoxy)pyridin-2-ylthio)-1-tosyl-1*H*-benzo[*d*] imidazole which was purified by column chromatography over silica gel using hexane-EtOAc (6:4) mixture as eluent.

### 3. Refinement

All the H atoms were fixed geometrically (C—H = 0.93–0.96 Å) and allowed to ride on their parent atoms with *U*_iso_(H) = 1.5*U*_eq_(C) for mehtyl H atoms and 1.2*U*_eq_(C) for the others.

### Crystal data

**Table:**

C_23_H_20_F_3_N_3_O_3_S_2_	*Z* = 2
*M**_r_* = 507.56	*F*(000) = 524
Triclinic, *P*1	*D*_x_ = 1.444 Mg m^−^^3^
Hall symbol: -P 1	Cu *K*α radiation, λ = 1.54178 Å
*a* = 9.1129 (6) Å	Cell parameters from 3779 reflections
*b* = 9.5116 (6) Å	θ = 3.3–64.3°
*c* = 13.7914 (8) Å	µ = 2.56 mm^−^^1^
α = 90.978 (3)°	*T* = 296 K
β = 101.749 (2)°	Block, colorless
γ = 93.449 (3)°	0.21 × 0.20 × 0.20 mm
*V* = 1167.72 (13) Å^3^	

### Data collection

**Table:**

Bruker X8 Proteum diffractometer	3779 independent reflections
Radiation source: Bruker MicroStar microfocus rotating anode	3301 reflections with *I* > 2σ(*I*)
Helios multilayer optics monochromator	*R*_int_ = 0.049
Detector resolution: 10.7 pixels mm^-1^	θ_max_ = 64.3°, θ_min_ = 3.3°
\φ and \ω scans	*h* = −10→10
Absorption correction: multi-scan (*SADABS*; Bruker, 2013)	*k* = −10→9
*T*_min_ = 0.615, *T*_max_ = 0.628	*l* = −15→16
13085 measured reflections	

### Refinement

**Table:**

Refinement on *F*^2^	Secondary atom site location: difference Fourier map
Least-squares matrix: full	Hydrogen site location: inferred from neighbouring sites
*R*[*F*^2^ > 2σ(*F*^2^)] = 0.050	H-atom parameters constrained
*wR*(*F*^2^) = 0.144	*w* = 1/[σ^2^(*F*_o_^2^) + (0.0866*P*)^2^ + 0.3269*P*] where *P* = (*F*_o_^2^ + 2*F*_c_^2^)/3
*S* = 1.08	(Δ/σ)_max_ = 0.001
3779 reflections	Δρ_max_ = 0.32 e Å^−^^3^
310 parameters	Δρ_min_ = −0.44 e Å^−^^3^
0 restraints	Extinction correction: *SHELXL97* (Sheldrick, 2008), FC^*^=KFC[1+0.001XFC^2^Λ^3^/SIN(2Θ)]^-1/4^
Primary atom site location: structure-invariant direct methods	Extinction coefficient: 0.034 (2)

### Special details

**Table:**

Geometry. Bond distances, angles *etc*. have been calculated using the rounded fractional coordinates. All su's are estimated from the variances of the (full) variance-covariance matrix. The cell e.s.d.'s are taken into account in the estimation of distances, angles and torsion angles
Refinement. Refinement on *F*^2^ for ALL reflections except those flagged by the user for potential systematic errors. Weighted *R*-factors *wR* and all goodnesses of fit *S* are based on *F*^2^, conventional *R*-factors *R* are based on *F*, with *F* set to zero for negative *F*^2^. The observed criterion of *F*^2^ > σ(*F*^2^) is used only for calculating -*R*-factor-obs *etc*. and is not relevant to the choice of reflections for refinement. *R*-factors based on *F*^2^ are statistically about twice as large as those based on *F*, and *R*-factors based on ALL data will be even larger.

### Fractional atomic coordinates and isotropic or equivalent isotropic displacement parameters (Å^2^)

**Table:**

	*x*	*y*	*z*	*U*_iso_*/*U*_eq_	
S1	0.12891 (6)	0.82368 (6)	0.42720 (4)	0.0417 (2)	
S2	0.02074 (7)	0.62948 (6)	0.21001 (4)	0.0407 (2)	
F1	0.4537 (4)	1.3138 (6)	1.0004 (3)	0.187 (2)	
F2	0.6843 (3)	1.3482 (4)	1.00123 (18)	0.1404 (13)	
F3	0.5875 (6)	1.1445 (5)	1.0016 (2)	0.197 (2)	
O1	0.4077 (2)	1.1690 (2)	0.82128 (13)	0.0587 (7)	
O2	−0.0894 (2)	0.6391 (2)	0.12112 (12)	0.0574 (6)	
O3	0.1428 (2)	0.7333 (2)	0.23364 (15)	0.0602 (7)	
N1	0.3136 (2)	1.0313 (2)	0.52852 (15)	0.0472 (7)	
N2	−0.0682 (2)	0.6339 (2)	0.30437 (14)	0.0397 (6)	
N3	−0.1048 (2)	0.6528 (2)	0.46116 (14)	0.0396 (6)	
C1	−0.1932 (2)	0.5419 (2)	0.30940 (17)	0.0377 (7)	
C2	−0.2849 (3)	0.4533 (3)	0.23902 (19)	0.0510 (8)	
C3	−0.4006 (3)	0.3771 (3)	0.2697 (2)	0.0596 (9)	
C4	−0.4223 (3)	0.3906 (3)	0.3663 (2)	0.0565 (9)	
C5	−0.3293 (3)	0.4789 (3)	0.4361 (2)	0.0500 (9)	
C6	−0.2124 (2)	0.5556 (3)	0.40671 (17)	0.0381 (7)	
C7	−0.0205 (2)	0.6966 (2)	0.40053 (16)	0.0360 (7)	
C8	0.1196 (3)	0.8619 (3)	0.55503 (17)	0.0412 (7)	
C9	0.2372 (2)	0.9781 (2)	0.59468 (17)	0.0388 (7)	
C10	0.2641 (2)	1.0241 (3)	0.69378 (17)	0.0403 (7)	
C11	0.3797 (3)	1.1290 (3)	0.72328 (18)	0.0417 (7)	
C12	0.4595 (3)	1.1839 (3)	0.65641 (19)	0.0464 (8)	
C13	0.4215 (3)	1.1312 (3)	0.5606 (2)	0.0498 (8)	
C14	0.1751 (3)	0.9647 (4)	0.7657 (2)	0.0601 (10)	
C15	0.5340 (3)	1.2630 (3)	0.8564 (2)	0.0569 (9)	
C16	0.5639 (5)	1.2654 (5)	0.9644 (3)	0.0892 (14)	
C17	0.0890 (2)	0.4606 (3)	0.21438 (16)	0.0382 (7)	
C18	0.1245 (3)	0.3915 (3)	0.30229 (18)	0.0453 (8)	
C19	0.1859 (3)	0.2627 (3)	0.3023 (2)	0.0546 (9)	
C20	0.2126 (3)	0.2022 (3)	0.2166 (3)	0.0589 (10)	
C21	0.1750 (4)	0.2735 (3)	0.1284 (2)	0.0635 (11)	
C22	0.1128 (3)	0.4017 (3)	0.12668 (19)	0.0535 (9)	
C23	0.2827 (5)	0.0626 (4)	0.2177 (4)	0.0963 (18)	
H2	−0.26980	0.44530	0.17450	0.0610*	
H3	−0.46500	0.31570	0.22500	0.0710*	
H4	−0.50170	0.33860	0.38440	0.0680*	
H5	−0.34450	0.48670	0.50060	0.0600*	
H8A	0.02080	0.89130	0.55870	0.0490*	
H8B	0.13810	0.77850	0.59400	0.0490*	
H12	0.53620	1.25400	0.67540	0.0560*	
H13	0.47500	1.16800	0.51510	0.0600*	
H14A	0.20290	1.01720	0.82750	0.0900*	
H14B	0.19570	0.86770	0.77640	0.0900*	
H14C	0.06990	0.97120	0.73930	0.0900*	
H15A	0.51420	1.35660	0.83230	0.0680*	
H15B	0.62040	1.23200	0.83280	0.0680*	
H18	0.10740	0.43130	0.36070	0.0540*	
H19	0.20980	0.21550	0.36130	0.0650*	
H21	0.19240	0.23390	0.07000	0.0760*	
H22	0.08690	0.44830	0.06750	0.0640*	
H23A	0.29340	0.02450	0.28260	0.1440*	
H23B	0.37970	0.07580	0.20090	0.1440*	
H23C	0.21960	−0.00150	0.17020	0.1440*	

### Atomic displacement parameters (Å^2^)

**Table:**

	*U*^11^	*U*^22^	*U*^33^	*U*^12^	*U*^13^	*U*^23^
S1	0.0434 (4)	0.0409 (4)	0.0391 (4)	−0.0068 (2)	0.0078 (2)	−0.0072 (2)
S2	0.0505 (4)	0.0388 (4)	0.0338 (3)	−0.0013 (3)	0.0122 (2)	−0.0017 (2)
F1	0.139 (3)	0.308 (6)	0.117 (2)	−0.066 (3)	0.069 (2)	−0.115 (3)
F2	0.122 (2)	0.206 (3)	0.0717 (15)	−0.084 (2)	0.0018 (13)	−0.0583 (17)
F3	0.259 (5)	0.185 (4)	0.094 (2)	−0.070 (3)	−0.066 (3)	0.061 (2)
O1	0.0533 (10)	0.0744 (14)	0.0421 (10)	−0.0183 (9)	0.0034 (8)	−0.0186 (9)
O2	0.0747 (12)	0.0635 (12)	0.0331 (9)	0.0219 (10)	0.0038 (8)	0.0065 (8)
O3	0.0721 (12)	0.0507 (11)	0.0633 (12)	−0.0208 (9)	0.0352 (10)	−0.0118 (9)
N1	0.0501 (11)	0.0462 (12)	0.0438 (11)	−0.0070 (9)	0.0098 (9)	−0.0036 (9)
N2	0.0426 (10)	0.0418 (11)	0.0339 (10)	−0.0063 (8)	0.0092 (8)	−0.0078 (8)
N3	0.0418 (10)	0.0427 (11)	0.0338 (10)	−0.0023 (8)	0.0087 (8)	−0.0032 (8)
C1	0.0346 (11)	0.0383 (13)	0.0386 (12)	−0.0019 (9)	0.0054 (9)	−0.0037 (9)
C2	0.0505 (14)	0.0557 (16)	0.0422 (13)	−0.0100 (12)	0.0037 (11)	−0.0098 (11)
C3	0.0480 (14)	0.0624 (18)	0.0596 (17)	−0.0157 (13)	−0.0031 (12)	−0.0074 (13)
C4	0.0434 (14)	0.0596 (18)	0.0630 (17)	−0.0142 (12)	0.0072 (12)	0.0062 (13)
C5	0.0453 (13)	0.0576 (17)	0.0474 (14)	−0.0037 (12)	0.0122 (11)	0.0035 (12)
C6	0.0349 (11)	0.0380 (13)	0.0402 (12)	0.0012 (9)	0.0051 (9)	−0.0012 (9)
C7	0.0382 (11)	0.0345 (12)	0.0337 (11)	0.0033 (9)	0.0041 (9)	−0.0052 (9)
C8	0.0421 (12)	0.0397 (13)	0.0391 (12)	−0.0046 (10)	0.0050 (10)	−0.0058 (10)
C9	0.0373 (11)	0.0373 (13)	0.0393 (12)	0.0038 (10)	0.0018 (9)	−0.0032 (9)
C10	0.0354 (11)	0.0415 (13)	0.0414 (13)	0.0004 (10)	0.0025 (9)	−0.0026 (10)
C11	0.0372 (12)	0.0434 (14)	0.0399 (12)	0.0016 (10)	−0.0017 (9)	−0.0068 (10)
C12	0.0426 (13)	0.0429 (14)	0.0496 (14)	−0.0042 (10)	0.0022 (11)	−0.0040 (11)
C13	0.0526 (14)	0.0480 (15)	0.0482 (14)	−0.0080 (12)	0.0125 (11)	−0.0008 (11)
C14	0.0582 (16)	0.074 (2)	0.0444 (15)	−0.0148 (14)	0.0081 (12)	−0.0046 (13)
C15	0.0489 (14)	0.0590 (18)	0.0558 (16)	−0.0104 (13)	−0.0002 (12)	−0.0147 (13)
C16	0.084 (2)	0.116 (3)	0.057 (2)	−0.039 (2)	0.0053 (18)	−0.025 (2)
C17	0.0359 (11)	0.0426 (13)	0.0349 (11)	0.0001 (9)	0.0051 (9)	−0.0004 (9)
C18	0.0425 (12)	0.0534 (16)	0.0389 (13)	−0.0009 (11)	0.0068 (10)	0.0041 (11)
C19	0.0399 (13)	0.0563 (17)	0.0666 (18)	0.0007 (12)	0.0081 (12)	0.0198 (14)
C20	0.0427 (14)	0.0437 (16)	0.093 (2)	0.0019 (11)	0.0202 (14)	0.0095 (15)
C21	0.0745 (19)	0.0541 (18)	0.0675 (19)	0.0144 (15)	0.0257 (15)	−0.0066 (14)
C22	0.0694 (17)	0.0555 (17)	0.0374 (13)	0.0129 (14)	0.0126 (12)	−0.0010 (11)
C23	0.093 (3)	0.050 (2)	0.160 (4)	0.0240 (19)	0.052 (3)	0.022 (2)

### Geometric parameters (Å, º)

**Table:**

S1—C7	1.742 (2)	C15—C16	1.458 (5)
S1—C8	1.814 (2)	C17—C18	1.378 (3)
S2—O2	1.4256 (18)	C17—C22	1.386 (3)
S2—O3	1.424 (2)	C18—C19	1.377 (4)
S2—N2	1.669 (2)	C19—C20	1.378 (5)
S2—C17	1.755 (3)	C20—C21	1.394 (5)
F1—C16	1.311 (6)	C20—C23	1.507 (5)
F2—C16	1.318 (6)	C21—C22	1.374 (4)
F3—C16	1.279 (6)	C2—H2	0.9300
O1—C11	1.366 (3)	C3—H3	0.9300
O1—C15	1.412 (3)	C4—H4	0.9300
N1—C9	1.344 (3)	C5—H5	0.9300
N1—C13	1.327 (3)	C8—H8A	0.9700
N2—C1	1.408 (3)	C8—H8B	0.9700
N2—C7	1.418 (3)	C12—H12	0.9300
N3—C6	1.396 (3)	C13—H13	0.9300
N3—C7	1.303 (3)	C14—H14A	0.9600
C1—C2	1.381 (3)	C14—H14B	0.9600
C1—C6	1.393 (3)	C14—H14C	0.9600
C2—C3	1.384 (4)	C15—H15A	0.9700
C3—C4	1.391 (4)	C15—H15B	0.9700
C4—C5	1.380 (4)	C18—H18	0.9300
C5—C6	1.387 (4)	C19—H19	0.9300
C8—C9	1.506 (3)	C21—H21	0.9300
C9—C10	1.396 (3)	C22—H22	0.9300
C10—C11	1.399 (4)	C23—H23A	0.9600
C10—C14	1.501 (4)	C23—H23B	0.9600
C11—C12	1.377 (4)	C23—H23C	0.9600
C12—C13	1.374 (4)		
			
C7—S1—C8	97.78 (12)	C18—C19—C20	121.4 (3)
O2—S2—O3	119.76 (12)	C19—C20—C21	118.7 (3)
O2—S2—N2	107.33 (10)	C19—C20—C23	121.0 (4)
O2—S2—C17	108.93 (11)	C21—C20—C23	120.3 (4)
O3—S2—N2	105.97 (11)	C20—C21—C22	120.8 (3)
O3—S2—C17	109.92 (11)	C17—C22—C21	119.1 (2)
N2—S2—C17	103.68 (10)	C1—C2—H2	122.00
C11—O1—C15	117.2 (2)	C3—C2—H2	122.00
C9—N1—C13	117.5 (2)	C2—C3—H3	119.00
S2—N2—C1	122.71 (16)	C4—C3—H3	119.00
S2—N2—C7	129.85 (15)	C3—C4—H4	119.00
C1—N2—C7	105.71 (17)	C5—C4—H4	119.00
C6—N3—C7	105.98 (18)	C4—C5—H5	121.00
N2—C1—C2	131.7 (2)	C6—C5—H5	121.00
N2—C1—C6	105.12 (19)	S1—C8—H8A	110.00
C2—C1—C6	123.2 (2)	S1—C8—H8B	110.00
C1—C2—C3	116.4 (2)	C9—C8—H8A	110.00
C2—C3—C4	121.2 (3)	C9—C8—H8B	110.00
C3—C4—C5	121.9 (3)	H8A—C8—H8B	108.00
C4—C5—C6	117.7 (2)	C11—C12—H12	121.00
N3—C6—C1	110.94 (19)	C13—C12—H12	121.00
N3—C6—C5	129.4 (2)	N1—C13—H13	118.00
C1—C6—C5	119.7 (2)	C12—C13—H13	118.00
S1—C7—N2	121.46 (15)	C10—C14—H14A	109.00
S1—C7—N3	126.22 (17)	C10—C14—H14B	109.00
N2—C7—N3	112.24 (18)	C10—C14—H14C	109.00
S1—C8—C9	108.03 (17)	H14A—C14—H14B	109.00
N1—C9—C8	115.2 (2)	H14A—C14—H14C	109.00
N1—C9—C10	123.44 (19)	H14B—C14—H14C	110.00
C8—C9—C10	121.35 (19)	O1—C15—H15A	110.00
C9—C10—C11	116.4 (2)	O1—C15—H15B	110.00
C9—C10—C14	122.4 (2)	C16—C15—H15A	110.00
C11—C10—C14	121.2 (2)	C16—C15—H15B	110.00
O1—C11—C10	115.5 (2)	H15A—C15—H15B	108.00
O1—C11—C12	123.8 (2)	C17—C18—H18	121.00
C10—C11—C12	120.8 (2)	C19—C18—H18	121.00
C11—C12—C13	117.5 (3)	C18—C19—H19	119.00
N1—C13—C12	124.4 (3)	C20—C19—H19	119.00
O1—C15—C16	108.1 (3)	C20—C21—H21	120.00
F1—C16—F2	106.4 (4)	C22—C21—H21	120.00
F1—C16—F3	106.5 (4)	C17—C22—H22	120.00
F1—C16—C15	112.6 (4)	C21—C22—H22	120.00
F2—C16—F3	106.4 (4)	C20—C23—H23A	109.00
F2—C16—C15	110.7 (3)	C20—C23—H23B	109.00
F3—C16—C15	113.7 (4)	C20—C23—H23C	109.00
S2—C17—C18	121.46 (19)	H23A—C23—H23B	110.00
S2—C17—C22	117.46 (19)	H23A—C23—H23C	109.00
C18—C17—C22	121.0 (3)	H23B—C23—H23C	109.00
C17—C18—C19	119.0 (2)		
			
C8—S1—C7—N2	176.89 (17)	N2—C1—C6—N3	0.4 (2)
C8—S1—C7—N3	0.4 (2)	N2—C1—C6—C5	179.2 (2)
C7—S1—C8—C9	−177.25 (16)	C2—C1—C6—N3	−179.6 (2)
O2—S2—N2—C1	53.2 (2)	C2—C1—C6—C5	−0.7 (4)
O2—S2—N2—C7	−143.98 (19)	C1—C2—C3—C4	0.3 (4)
O3—S2—N2—C1	−177.76 (17)	C2—C3—C4—C5	−0.7 (4)
O3—S2—N2—C7	−14.9 (2)	C3—C4—C5—C6	0.4 (4)
C17—S2—N2—C1	−62.02 (19)	C4—C5—C6—N3	178.9 (3)
C17—S2—N2—C7	100.8 (2)	C4—C5—C6—C1	0.3 (4)
O2—S2—C17—C18	−143.1 (2)	S1—C8—C9—N1	3.6 (2)
O2—S2—C17—C22	40.0 (2)	S1—C8—C9—C10	−175.61 (17)
O3—S2—C17—C18	83.9 (2)	N1—C9—C10—C11	−1.4 (3)
O3—S2—C17—C22	−93.1 (2)	N1—C9—C10—C14	178.8 (2)
N2—S2—C17—C18	−29.0 (2)	C8—C9—C10—C11	177.7 (2)
N2—S2—C17—C22	154.01 (19)	C8—C9—C10—C14	−2.0 (4)
C15—O1—C11—C10	173.1 (2)	C9—C10—C11—O1	−177.8 (2)
C15—O1—C11—C12	−5.6 (4)	C9—C10—C11—C12	1.0 (4)
C11—O1—C15—C16	−166.7 (3)	C14—C10—C11—O1	2.0 (4)
C13—N1—C9—C8	−178.1 (2)	C14—C10—C11—C12	−179.3 (3)
C13—N1—C9—C10	1.1 (3)	O1—C11—C12—C13	178.4 (3)
C9—N1—C13—C12	−0.3 (4)	C10—C11—C12—C13	−0.3 (4)
S2—N2—C1—C2	−13.5 (3)	C11—C12—C13—N1	−0.1 (4)
S2—N2—C1—C6	166.61 (16)	O1—C15—C16—F1	−63.2 (5)
C7—N2—C1—C2	−179.9 (2)	O1—C15—C16—F2	177.9 (3)
C7—N2—C1—C6	0.2 (2)	O1—C15—C16—F3	58.1 (5)
S2—N2—C7—S1	17.2 (3)	S2—C17—C18—C19	−176.2 (2)
S2—N2—C7—N3	−165.81 (16)	C22—C17—C18—C19	0.7 (4)
C1—N2—C7—S1	−177.72 (14)	S2—C17—C22—C21	176.0 (2)
C1—N2—C7—N3	−0.8 (2)	C18—C17—C22—C21	−1.1 (4)
C7—N3—C6—C1	−0.8 (3)	C17—C18—C19—C20	0.1 (4)
C7—N3—C6—C5	−179.5 (3)	C18—C19—C20—C21	−0.5 (4)
C6—N3—C7—S1	177.75 (17)	C18—C19—C20—C23	178.8 (3)
C6—N3—C7—N2	1.0 (2)	C19—C20—C21—C22	0.1 (5)
N2—C1—C2—C3	−179.5 (2)	C23—C20—C21—C22	−179.2 (3)
C6—C1—C2—C3	0.4 (4)	C20—C21—C22—C17	0.7 (5)

### Hydrogen-bond geometry (Å, º)

Cg1 is the centroid of the imidazole ring.

**Table:**

*D*—H···*A*	*D*—H	H···*A*	*D*···*A*	*D*—H···*A*
C2—H2···F2^i^	0.93	2.49	3.363 (4)	156
C15—H15*B*···O3^ii^	0.97	2.52	3.420 (3)	155
C18—H18···N3^iii^	0.93	2.60	3.336 (3)	136
C18—H18···*Cg*1	0.93	2.83	3.463 (3)	126

Symmetry codes: (i) *x*−1, *y*−1, *z*−1; (ii) −*x*+1, −*y*+2, −*z*+1; (iii) −*x*, −*y*+1, −*z*+1.
